# DO I GET BY WITH A LITTLE HELP FROM MY FRIENDS? FINDINGS FROM THE IOWA UNMARRIED SURVIVORS STUDY

**DOI:** 10.1093/geroni/igac059.2639

**Published:** 2022-12-20

**Authors:** Robert Hensley, Alex Bishop, Peter Martin

**Affiliations:** Southeast Community College, Lincoln, Nebraska, United States; Oklahoma State University, Stillwater, Oklahoma, United States; Iowa State University, Ames, Iowa, United States

## Abstract

The purpose of this study was to analyze the role that selected personality variables (Neuroticism, Agreeableness, Conscientiousness, Extroversion, and Openness), social support, and coping played in loneliness of 227 (M = 78.2, SD = 8.1) participants in the Iowa Unmarried Survivors Study. This study is a sample of never married, divorced, and widowed participants. Blocked multiple regression analyses were used in this study. In the first block, age, gender, ethnicity, past schooling, current marital status, total illnesses, and childhood poverty were included. The second block contained the aforementioned personality variables, and the third block included both social support and coping. Results indicated that both Neuroticism and Extraversion were significant predictors of loneliness, β = .266, p < .00, β = -.294, p < .05, and β = .27, p < .00, respectively. In short, the greater the neuroticism, the higher the score in loneliness. Moreover, the lower the level of extraversion, the higher the score in loneliness. In addition, social support served as a significant predictor of loneliness, β = -.412, p < .00. The more social support in the participants’ lives, the lower level of loneliness. Finally, coping predicted loneliness. In essence, the more loneliness in their lives, the greater the use of coping mechanisms utilized. This model explained 58% of the variance in loneliness scores. Navigating through late adulthood as an unmarried survivor presents a host of challenges, and these results add to our understanding of the link between personality, social support, coping, and loneliness in late life.

